# Supplementary material for: BCL7A and BCL7B potentiate SWI/SNF-complex-mediated chromatin accessibility to regulate gene expression and vegetative phase transition in plants
Source: Nat Commun. 2024 Jan 31;15:935. doi: 10.1038/s41467-024-45250-x (PMC10830565; doi:10.1038/s41467-024-45250-x)
Supplement: Supplementary file 1 — Supplementary Information [file 41467_2024_45250_MOESM1_ESM.pdf]

Supplementary Information for

BCL7A and BCL7B potentiate SWI/SNF-complex-mediated chromatin accessibility to regulate gene expression and vegetative phase transition in plants

Yawen Lei[1, #], Yaoguang Yu[1, #], Wei Fu[1], Tao Zhu[1], Caihong Wu[1], Zhihao Zhang[1], Zewang Yu[1], Xin Song[1], Jianqu Xu[1], Zhenwei Liang[1], Peitao Lü[2], Chenlong Li[1*]

[1] State Key Laboratory of Biocontrol and Guangdong Key Laboratory of Plant Resources, School of Life Sciences, Sun Yat-sen University, Guangzhou, 510275, China

[2] College of Horticulture, FAFU-UCR Joint Center for Horticultural Biology and Metabolomics, Haixia Institute of Science and Technology, Fujian Agriculture and Forestry University, Fuzhou 350002, China

[#] The authors contributed equally to this work

* Corresponding author: Chenlong Li (lichlong3@mail.sysu.edu.cn)

This PDF file includes: 16 Supplementary Figures.

a

IP: BRM-GFP + mass spectrometry data from published data (Li .et al 2016)

Protein	Bio Repeats	Number of Unique peptide	Peptide Coverage (%)
BCL7A	IP1	6	21.5
	IP2	6	32.91
BCL7B	IP1	22	78.0
	IP2	14	73.2

IP: p35S:GFP + mass spectrometry data from published data (Li .et al 2016)

Protein	Bio Repeats	Number of Unique peptide	Peptide Coverage (%)
BCL7A	IP1	0	0
	IP2	0	0
BCL7B	IP1	0	0
	IP2	0	0

b

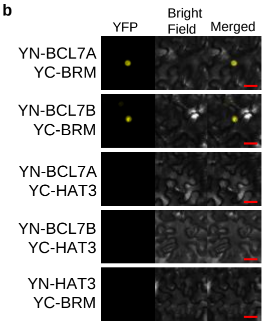

Supplementary Fig. 1 | BCL7A/B interact with BRM. a, Summary of the peptides of BCL7A and BCL7B identified by mass spectrometry from an anti-GFP purification of a BRM-GFP stably expressed line. Two biological replicates are shown. A *p35S:GFP* line was used as a negative control. **b**, Bimolecular fluorescence complementation assay (BiFC) showing that BCL7A and BCL7B interact with BRM in *planta*. A nuclear-localized protein *HAT3* was used as a negative control. Scale bars, 20 µm.

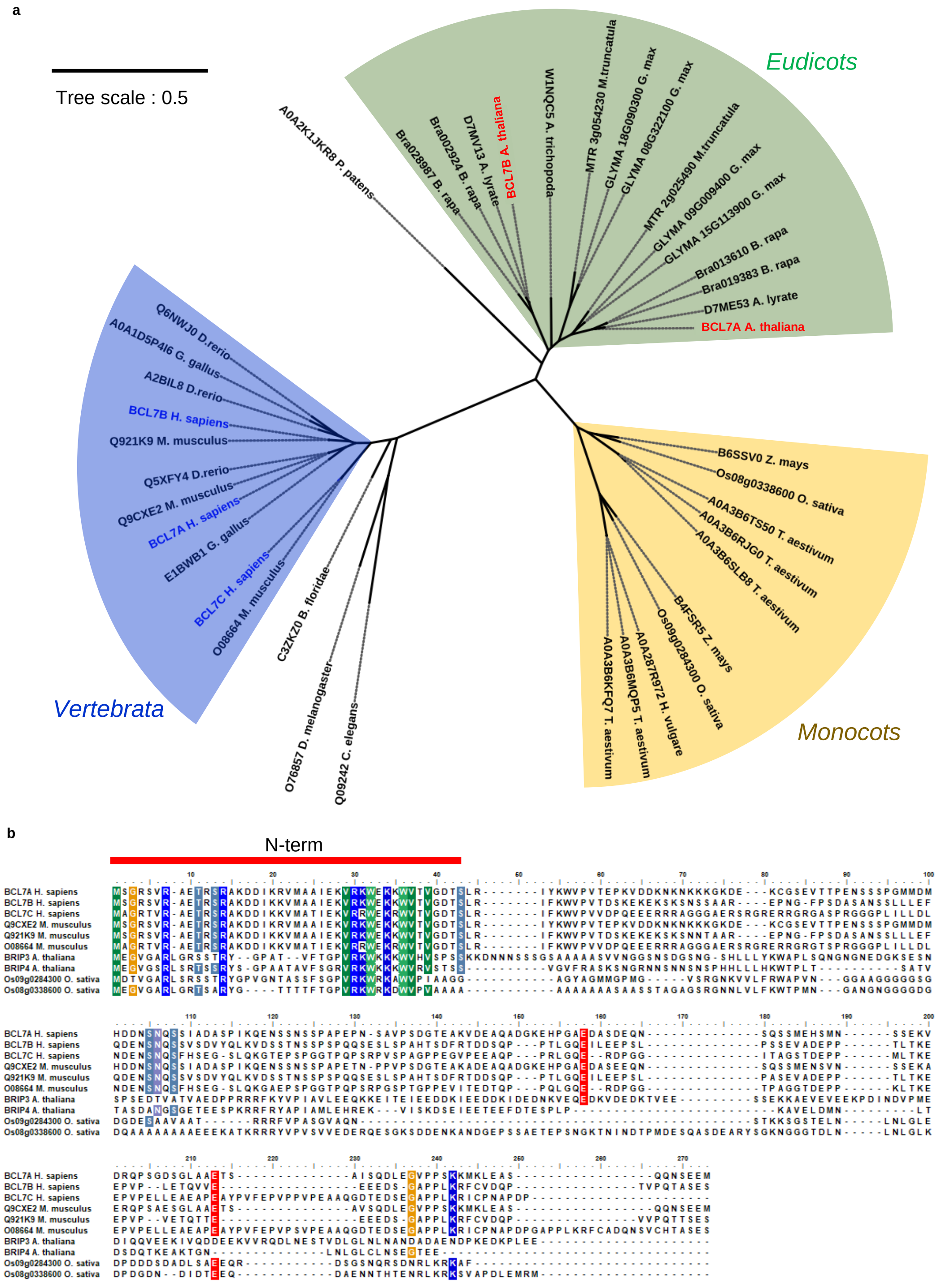

Supplementary Fig. 2 | Phylogenetic analysis of BCL7A/B homologs among different species. a, The phylogenetic tree was constructed using the amino-acid sequences of BCL7A/B and their homologous proteins from different species, including *Eudicots*, *Monocots* and *Vertebrata*. The scale length in the tree is indicated. **b**, The alignment of the sequences of BCL7A/B proteins and their homologous proteins in human, mouse and rice.

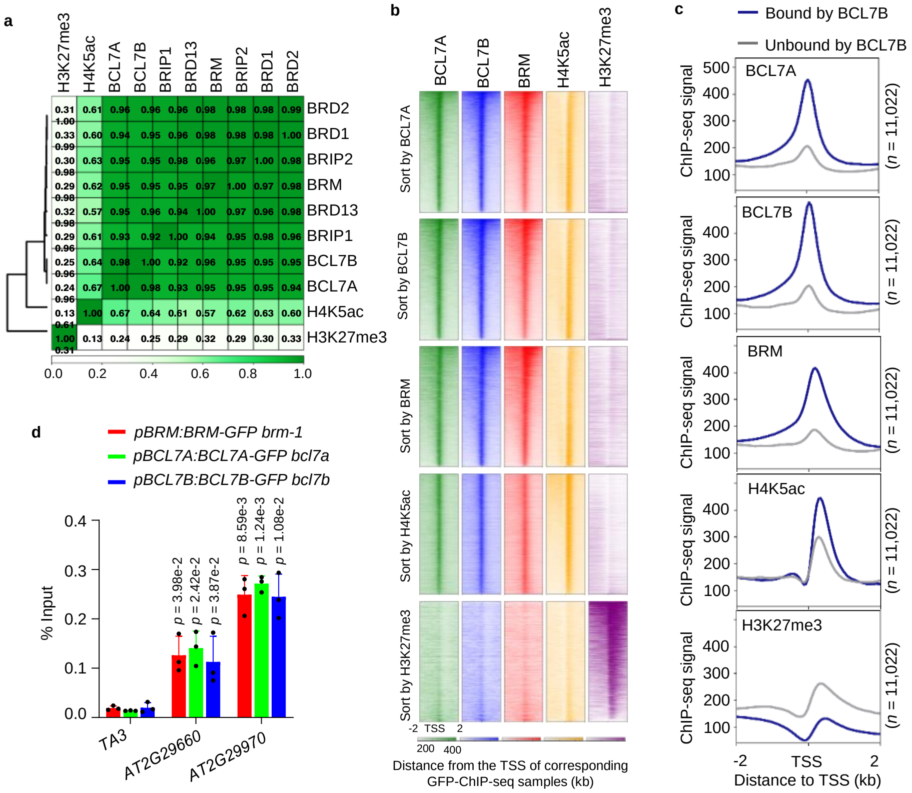

Supplementary Fig. 3 | BCL7A/B co-localize with BRM genome-wide. a, Matrix depicting spearman correlation coefficients between ChIP-seq datasets, calculated using the bin mode (bin size = 10,000). **b**, Heat map representations of ChIP-seq of BCL7A/B, BRM and histone modifications (H4K5ac and H3K27me3). Rank order is from highest to lowest of each ChIP-seq dataset signal. $\log_2$ enrichment was normalized to reads per genome coverage. Read counts per gene were averaged in 50-nucleotide (nt) bins. **c**, Average intensity of BCL7A, BCL7B, BRM, and histone modifications (H4K5ac and H3K27me3) ChIP-seq reads at BCL7B-bound genes versus BCL7B-unbound genes. Read counts per gene were summed in 50-nt bins. **d**, ChIP-qPCR showing occupancy by BRM-GFP, BCL7A-GFP and BCL7B-GFP at representative genes. *TA3* locus was used as the negative control. Error bars are presented as mean ± s.d. from three biological replicates. *P* values were calculated with the two-tailed Student's t-test.

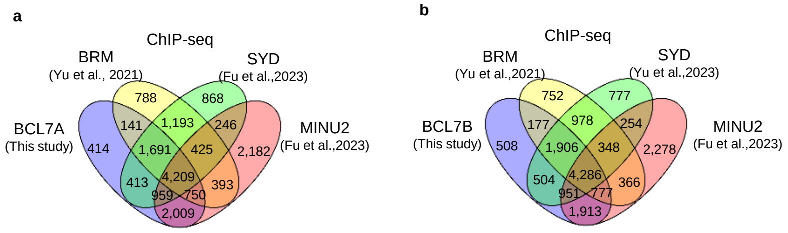

Supplementary Fig. 4 | BCL7A/B co-localize with SYD and MINU genome-wide. a, Venn diagrams displaying statistically significant overlaps among genes occupied by BCL7A, BRM, SYD, and MINU2. **b**, Venn diagrams displaying statistically significant overlaps among genes occupied by BCL7B, BRM, SYD, and MINU2.

a

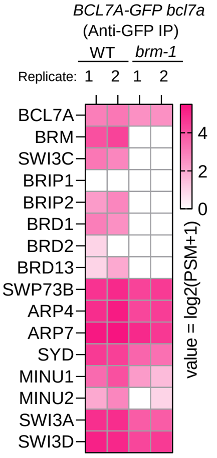

b

Unique peptides of BCL7A-GFP (IP: GFP)

protein	BCL7A-GFP		BCL7A-GFP *brm-1*	
	IP1	IP2	IP1	IP2
BCL7A (Bait)	5	5	4	3
BRM	16	19	0	0
SWI3C	7	5	0	0
BRIP1	0	0	0	0
BRIP2	4	6	0	0
BRD1	7	5	0	0
BRD2	1	0	0	0
BRD13	1	3	0	0
SWP73B	17	21	16	18
ARP4	15	19	13	14
ARP7	15	17	18	14
SYD	22	19	11	9
MINU1	8	13	4	2
MINU2	1	4	0	1
SWI3A	22	19	12	11
SWI3D	29	26	18	20

c

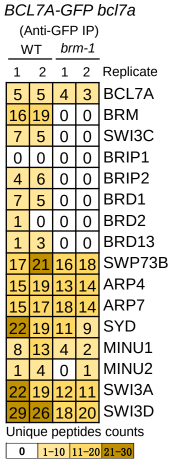

d

protein	WT	
	IP1	IP2
BCL7A	0	0
BCL7B	0	0
BRM	0	0
SWI3C	0	0
BRIP1	0	0
BRIP2	0	0
BRD1	0	0
BRD2	0	0
BRD13	0	0
SWP73B	0	0
ARP4	0	0
ARP7	0	0
SYD	0	0
MINU1	0	0
MINU2	0	0
SWI3A	0	0
SWI3D	0	0

e

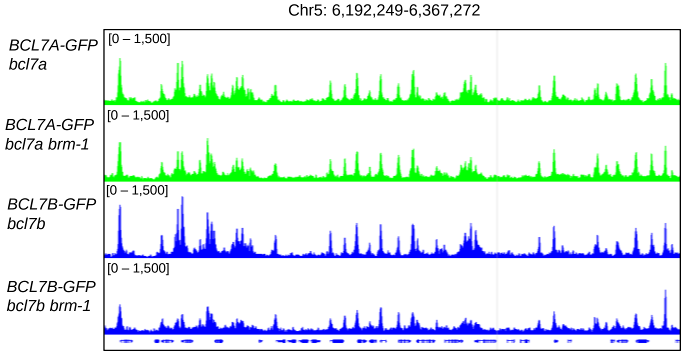

Supplementary Fig. 5 | BCL7A/B require BRM for assembling into the SWI/SNF complexes at target chromatin. a, Heat map showing the log_2(PSM+1) values of BRM-SWI/SNF complex subunits identified by IP-MS in *BCL7A-GFP* transgenic line under WT or *brm-1* mutant background. PSM: peptide spectrum match value. **b**, Unique peptide number of BRM-SWI/SNF complex subunits identified in *BCL7A-GFP bcl7a* and *BCL7A-GFP bcl7a brm-1*. Note: SYD, MINU1, MINU2, SWI3A, and SWI3D are not subunits of BRM-SWI/SNF complexes. **c**, Heat map showing the unique peptide number of BRM-SWI/SNF complex subunits identified by IP-MS in *BCL7A-GFP* transgenic line under WT or *brm-1* mutant background. **d**, Summary of the peptides of indicated proteins identified by mass spectrometry from WT. Two biological replicates are shown. **e**, IGV views of BCL7A or BCL7B occupancy at part of chromosome 5 in WT and *brm-1* background.

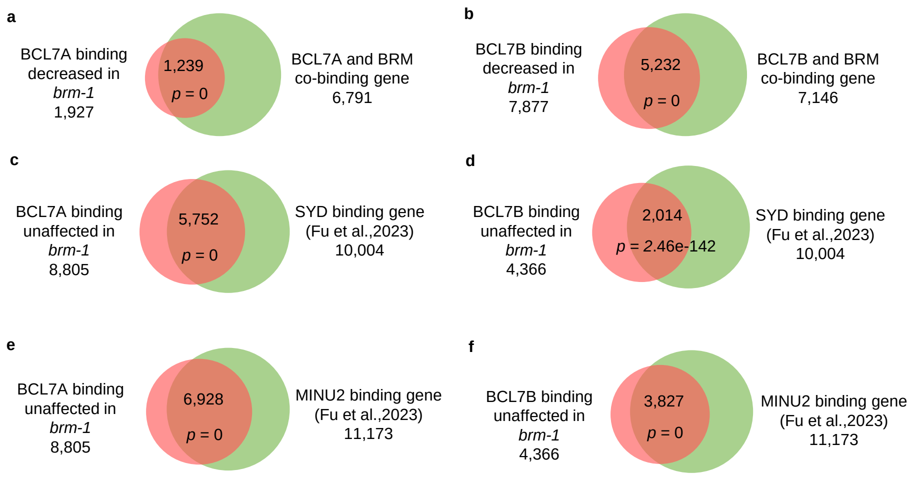

Supplementary Fig. 6 | The binding of BCL7A/B may be regulated by BRM, SYD, or MINU. **a** and **b**, Venn diagrams displaying statistically significant overlaps between the genes where BCL7A (a) or BCL7B (b) binding signal reduced in *brm-1* and those co-targeted by BRM/BCL7. **c** and **d**, Venn diagrams displaying statistically significant overlaps between the genes where BCL7A (c) or BCL7B (d) binding signal unaffected in *brm-1* and those targeted by SYD. **e** and **f**, Venn diagrams displaying statistically significant overlaps between the genes where BCL7A (e) or BCL7B (f) binding signal unaffected in *brm-1* and those targeted by MINU2. *P* values were calculated by the hypergeometric test.

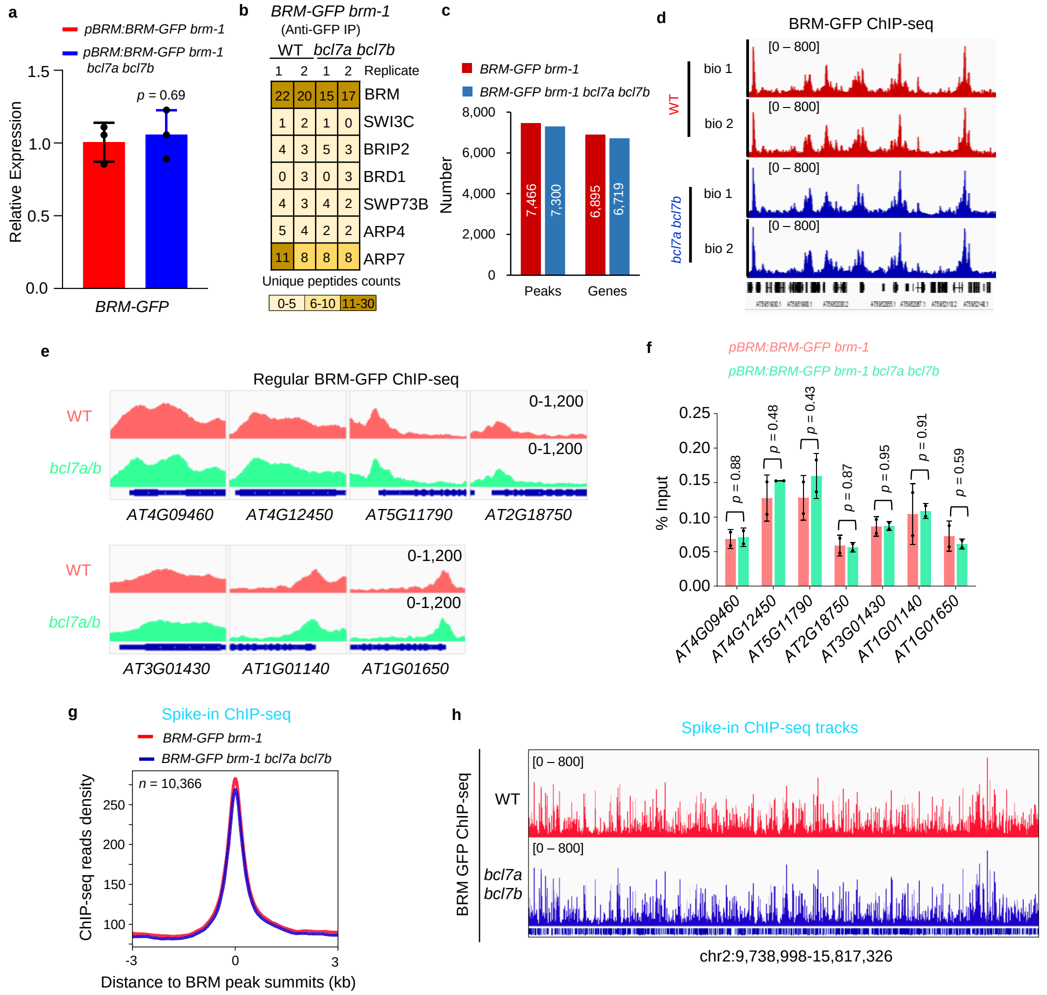

Supplementary Fig. 7 | Mutations in BCL7A/B do not affect BRM occupancy genome-wide. a, The mRNA levels of *BRM-GFP* in WT and *bcl7a bcl7b* background. *ACTIN2* was amplified as an internal control. Error bars are presented as mean ± s.d. from three biological replicates. *P* values were calculated with the two-tailed Student's t-test. **b**, Unique peptide number of SWI/SNF complex subunits identified in *BRM-GFP brm-1* and *BRM-GFP brm-1 bcl7a bcl7b*. **c**, Histogram showing the numbers of BRM binding sites (peaks or genes) in the WT and *bcl7a bcl7b* background. **d**, IGV views of BRM occupancy at selected loci in WT and *bcl7a bcl7b* background. **e**. IGV screenshots showing BRM occupancy on selected loci in the WT and *bcl7a bcl7b* background. **f**. Validation of ChIP-seq signals by ChIP-qPCR. Mean ± s.d. from two biological replicates. *P* values were calculated with the two-tailed Student's t-test. **g-h**, Metagene plot and IGV screenshots (**f**) representation of the mean density BRM occupancy at all BRM binding sites in the WT and *bcl7a bcl7b* background, as determined by spike-in ChIP-seq.

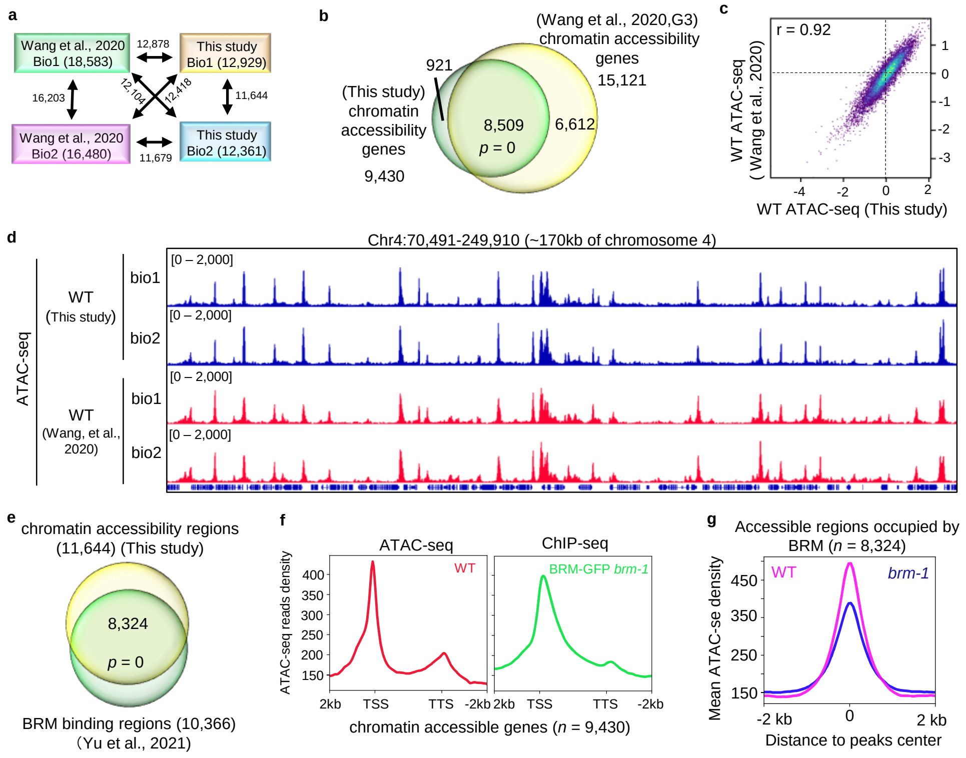

Supplementary Fig. 8 | Arabidopsis BRM directly regulates genome-wide chromatin accessibility. a, The overlap of peaks among the data used in this study and the published data. **b**, Venn diagram showing accessible genes in WT between this study and the published data. **c**, Scatter plot showing the correlation of ATAC-seq data between this study and the published data. *R* values are indicated. **d**, Genome browser snapshot of ATAC-seq data along a 170 kb stretch of chromosome 4 from this study and the published data. **e,** Venn diagram showing statistically significant overlaps between the chromatin accessibility regions and the BRM binding regions, according to the hypergeometric test. **f**, Metagene plots showing the mean density of ATAC-seq signals and BRM occupancy at accessible loci in WT. **g**, Metagene plot reflecting that the accessible regions occupied by BRM showed substantially reduced accessibility in *brm-1* null mutant compared with WT.

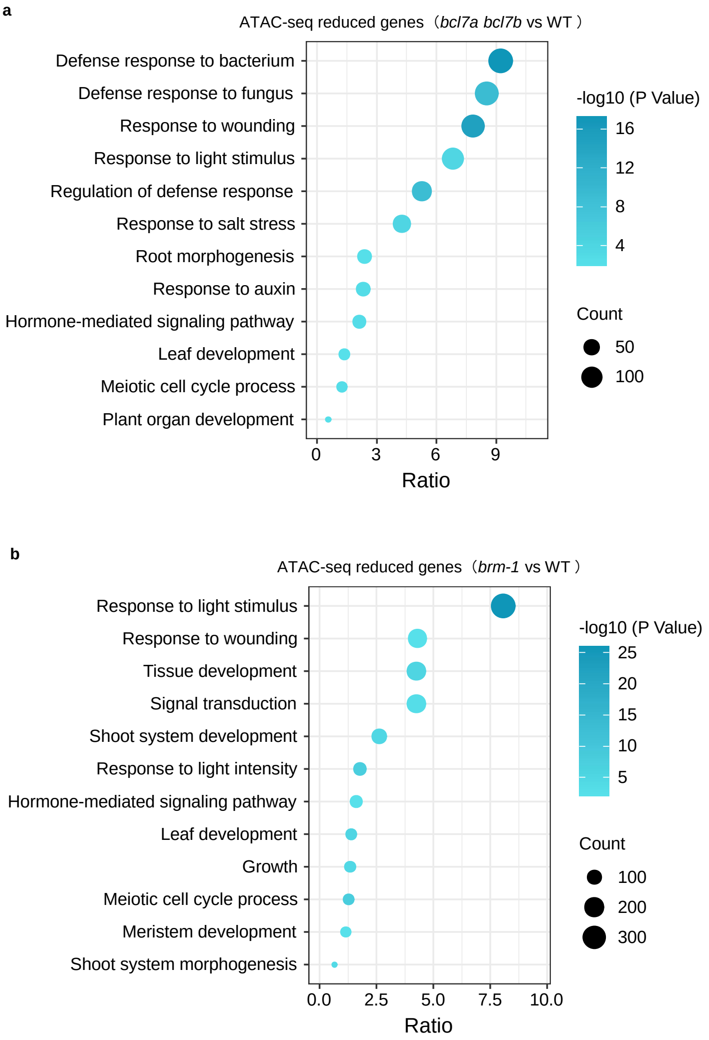

Supplementary Fig. 9 | Genes with reduced accessibility in *bcl7a bcl7b* and those in *brm-1* presented similar GO distribution. a, Gene ontology analysis of genes that reduced chromatin accessibility in *bcl7a bcl7b* mutant. **b**, Gene ontology analysis of genes with reduced chromatin accessibility in *brm-1* mutant.

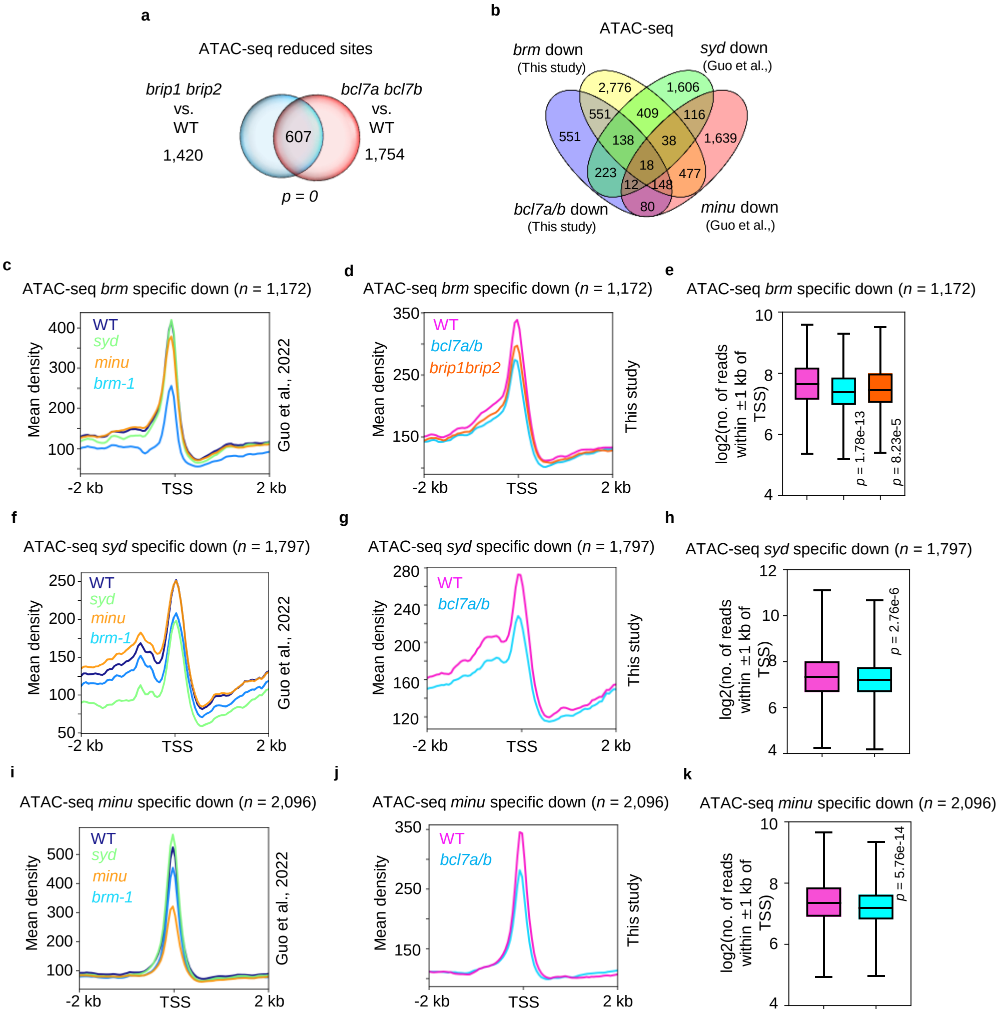

Supplementary Fig. 10 | BCL7A/B are likely involved in regulating the accessibility maintained by the SAS- and MAS-complexes. a, Venn diagram showing overlap between the genes that reduced accessibility in *bcl7a bcl7b* compared with WT and those in *brip1 brip2*. *P* values were calculated by the hypergeometric test. **b**, Venn diagram showing overlap between the genes that reduced accessibility in *bcl7a bcl7b* compared with WT and those in *brm-1*, *syd-5* and *minu1 minu2* mutants. **c**, Profile plots showing the accessibility signals in the indicated SWI/SNF mutants at the genes with decreased accessibility specifically identified in *brm* (Guo et al., 2022). **d-e**, Profile plots and box-plots showing the accessibility signals in the indicated mutants at the genes with decreased accessibility specifically identified in *brm*. **f**, Profile plots showing the accessibility signals in the indicated SWI/SNF mutants at the genes with decreased accessibility specifically identified in *syd* (Guo et al., 2022). **g-h**, Profile plots and box-plots showing the accessibility signals in the indicated mutants at the genes with decreased accessibility specifically identified in *syd*. **i**, Profile plots showing the accessibility signals in the indicated SWI/SNF mutants at the genes with decreased accessibility specifically identified in *minu* (Guo et al., 2022). **j-k**, Profile plots and box-plots showing the accessibility signals in the indicated mutants at the genes with decreased accessibility specifically identified in *minu*. In box plots (e, h, k), centre line and bounds of box represent median value and the interquartile range (IQR), respectively. Whiskers extend within 1.5 times the IQR. *P* values were calculated with the two-tailed Student's t-test.

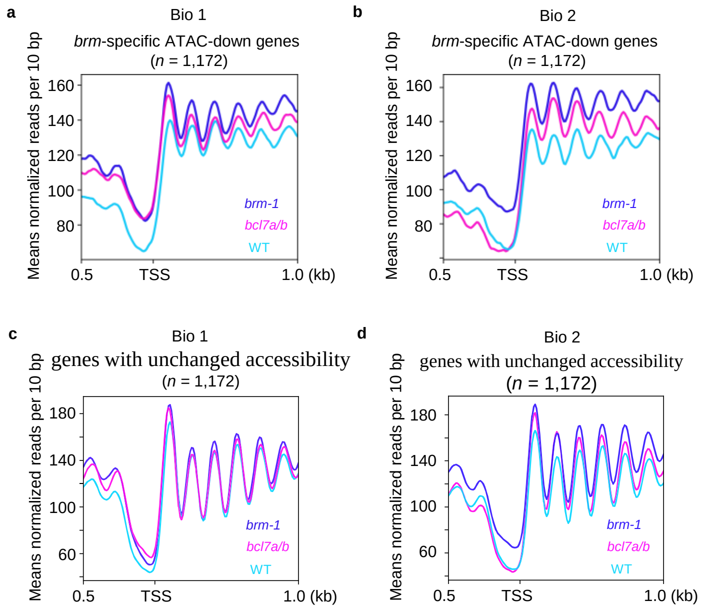

Supplementary Fig. 11 | BCL7A/B and BRM regulate nucleosome occupancy around TSS. a and **b**, Profile plots showing the average MNase-seq signals of WT, *bcl7a bcl7b*, and *brm-1* around TSS of the genes with decreased accessibility specifically identified in *brm*. **c** and **d**, Profile plots showing the average MNase-seq signals of WT, *bcl7a bcl7b*, and *brm-1* around TSS of the selected genes with unchanged accessibility in *brm*.

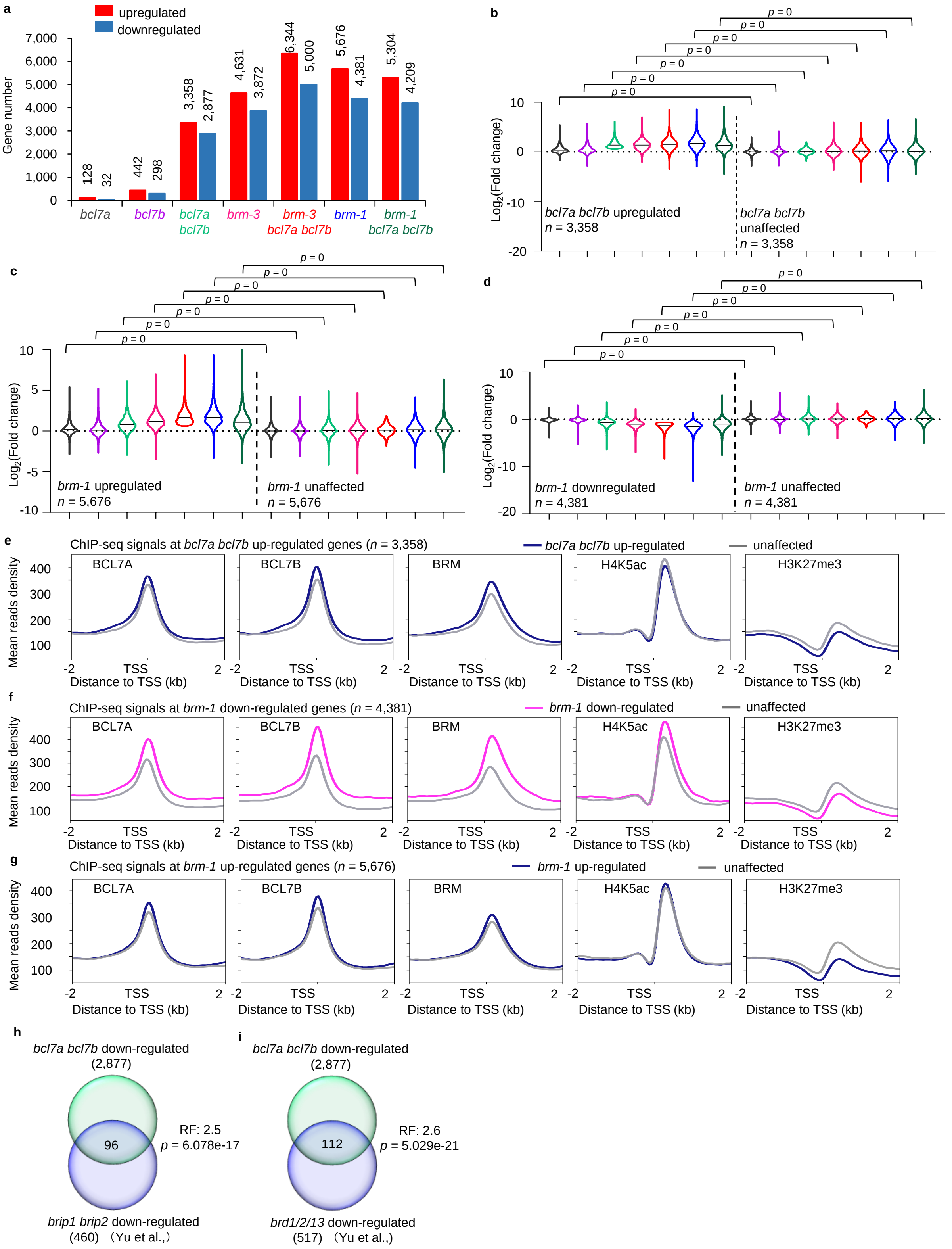
a
upregulated
downregulated
Gene number
7,000
6,000
5,000
4,000
3,000
2,000
1,000
0
128
32
442
298
3,358
2,877
4,631
3,872
6,344
5,000
5,676
4,381
5,304
4,209
bcl7a
bcl7b
bcl7a bcl7b
brm-3
brm-3 bcl7a bcl7b
brm-1
brm-1 bcl7a bcl7b
b
p = 0
Log2(Fold change)
10
0
-10
-20
bcl7a bcl7b upregulated
n = 3,358
bcl7a bcl7b unaffected
n = 3,358
c
p = 0
Log2(Fold change)
10
5
0
-5
-10
brm-1 upregulated
n = 5,676
brm-1 unaffected
n = 5,676
d
p = 0
Log2(Fold change)
10
0
-10
-20
brm-1 downregulated
n = 4,381
brm-1 unaffected
n = 4,381
e
ChIP-seq signals at bcl7a bcl7b up-regulated genes (n = 3,358)
bcl7a bcl7b up-regulated
unaffected
Mean reads density
400
300
200
100
BCL7A
BCL7B
BRM
H4K5ac
H3K27me3
-2
TSS
2
Distance to TSS (kb)
f
ChIP-seq signals at brm-1 down-regulated genes (n = 4,381)
brm-1 down-regulated
unaffected
Mean reads density
400
300
200
100
BCL7A
BCL7B
BRM
H4K5ac
H3K27me3
-2
TSS
2
Distance to TSS (kb)
g
ChIP-seq signals at brm-1 up-regulated genes (n = 5,676)
brm-1 up-regulated
unaffected
Mean reads density
400
300
200
100
BCL7A
BCL7B
BRM
H4K5ac
H3K27me3
-2
TSS
2
Distance to TSS (kb)
h
bcl7a bcl7b down-regulated
(2,877)
96
RF: 2.5
p = 6.078e-17
brip1 brip2 down-regulated
(460) （Yu et al.,）
i
bcl7a bcl7b down-regulated
(2,877)
112
RF: 2.6
p = 5.029e-21
brd1/2/13 down-regulated
(517) （Yu et al.,)

Supplementary Fig. 12 | BCL7A/B and BRM co-regulate gene expression. a, Summary of up- and down-regulated genes in different mutants compared to WT. The number of up- and down-regulated genes were shown. **b**, Violin plots depicting the $\log_2$(fold change) in RNA-seq for *bcl7a bcl7b* upregulated and unaffected genes in various mutants. *p* values were determined by the two-tailed Mann-Whitney *U* test. **c**, Violin plots depicting the $\log_2$(fold change) in RNA-seq for *brm-1* upregulated and unaffected genes in various mutants. *p* values were determined by the two-tailed Mann-Whitney *U* test. **d**, Violin plots depicting the $\log_2$(fold change) in RNA-seq for *brm-1* downregulated and unaffected genes in various mutants. *p* values were determined by the two-tailed Mann-Whitney *U* test. **e**, Average binding signals of the indicated ChIP-seq at *bcl7a bcl7b*-upregulated or *bcl7a bcl7b*-unaffected genes. Read counts per gene were summed in 50-nt bins. **f,** Average binding signals of the indicated ChIP-seq at *brm-1*-downregulated or *brm-1*-unaffected genes. **g,** Average binding signals of the indicated ChIP-seq at *brm-1*-upregulated or *brm-1*-unaffected genes. **h-i**, Venn diagrams showing statistically significant overlaps between genes down-regulated *in bcl7a bcl7b* and those in *brip1 brip2* or *brd1/2/13*, according to the hypergeometric test.

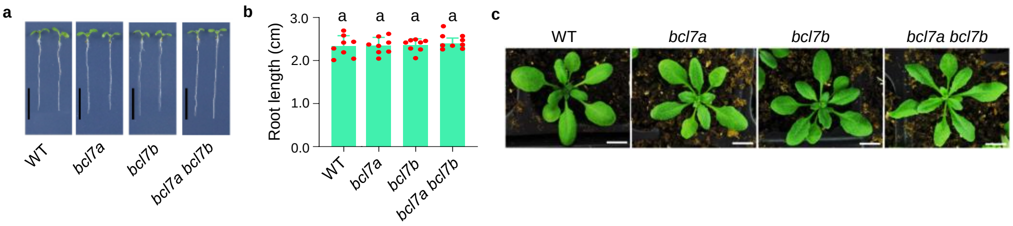

Supplementary Fig. 13 | Phenotype of *bcl7a*, *bcl7b* and *bcl7a bcl7b* mutant seedlings. a, Root phenotype of 7-day-old seedlings under LD condition. Scale bars, 1 cm. **b**, Root length of different 7-day-old seedlings. Lowercase letters indicate significant differences between genetic backgrounds, as determined by the *post hoc* Tukey's HSD test. Data are presented as mean ± s.d.; $n = 8$. **c**, Leaf phenotype of 21-day-old seedlings under LD condition. Scale bars, 1 cm.

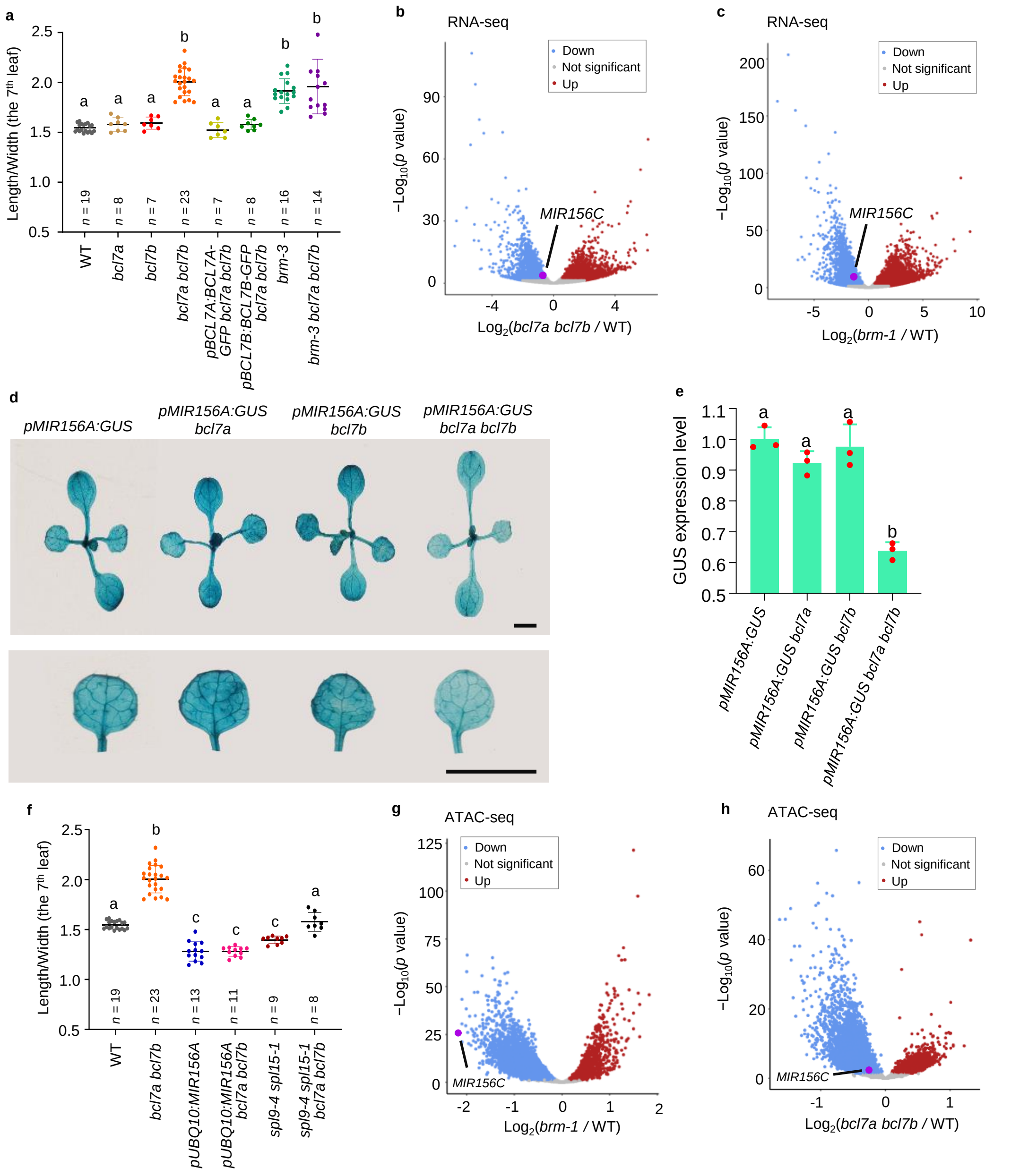

Supplementary Fig. 14 | BRM and BCL7A/B regulate the chromatin accessibility of *MIR156A/C*. a, The length/width ratio of the 7th leaf of indicated plant materials. '*n* =' indicates the number of plants that were used. Data are presented as mean ± s.d. Lower case letters indicate statistical significance determined by the *post hoc* Tukey's HSD test. **b-c**, Volcano plots showing differentially expressed genes in *bcl7a bcl7b* (**b**) or *brm-1* (**c**), determined by RNA-seq. *MIR156C* was significantly downregulated. **d**, GUS staining of *pMIR156A:GUS* in 14-day-old WT, *bcl7a*, *bcl7b*, and *bcl7a bcl7b* seedlings. **e**, The mRNA levels of *GUS* in WT, *bcl7a*, *bcl7b*, and *bcl7a bcl7b* seedlings. *ACTIN2* was amplified as an internal control. Error bars represent mean ± s.d. from three biological replicates. Lowercase letters indicate statistical significance determined by the *post hoc* Tukey's HSD test. **f**, The length/width ratio of the 7th leaf of indicated plant materials. Lowercase letters indicate statistical significance determined by the *post hoc* Tukey's HSD test. **g-h**, Volcano plots showing accessibility changed genes in *bcl7a bcl7b* (**g**) or *brm-1* (**h**). Pink dots indicate *MIR156C*.

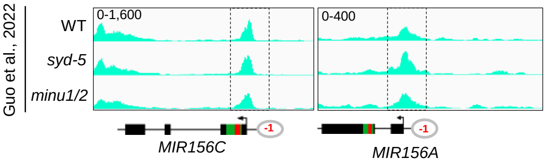

Supplementary Fig. 15 | Examples of ATAC-seq tracks at *MIR156A/C* loci in the WT, *syd-5*, and *minu1 minu2* mutants.

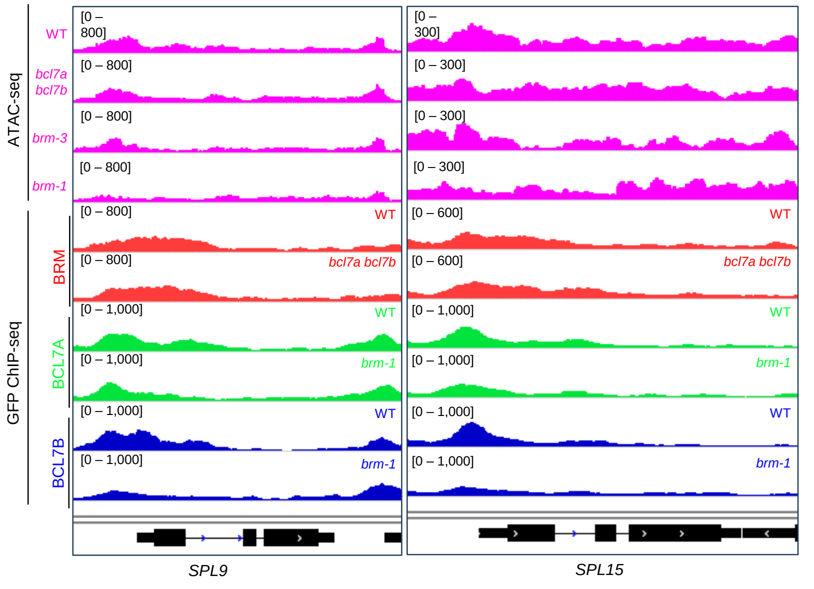

Supplementary Fig. 16 | IGV views of ATAC-seq and ChIP-seq signals on *SPL9* and *SPL15* in the indicated plant materials.
