# Supplementary material for: BCL7A and BCL7B potentiate SWI/SNF-complex-mediated chromatin accessibility to regulate gene expression and vegetative phase transition in plants
Source: Nat Commun. 2024 Jan 31;15:935. doi: 10.1038/s41467-024-45250-x (PMC10830565; doi:10.1038/s41467-024-45250-x)
Supplement: Supplementary file 3 — Description of Additional Supplementary Files [file 41467_2024_45250_MOESM3_ESM.pdf]

Description of Additional Supplementary Files

Supplementary Data Legends

Supplementary Data 1: Full list of Arabidopsis proteins identified by mass spectrometry analysis in this study.

Supplementary Data 2: Summary of mapped reads for ATAC-seq.

Supplementary Data 3: List of genes mis-regulated in different mutants used in this study.

Supplementary Data 4: Oligonucleotides used in this study.

Supplementary Data 5: Summary of mapped reads for ChIP-seq.
