# Supplementary material for: BCL7A and BCL7B potentiate SWI/SNF-complex-mediated chromatin accessibility to regulate gene expression and vegetative phase transition in plants
Source: Nat Commun. 2024 Jan 31;15:935. doi: 10.1038/s41467-024-45250-x (PMC10830565; doi:10.1038/s41467-024-45250-x)
Supplement: Supplementary file 9 — Reporting Summary [file 41467_2024_45250_MOESM9_ESM.pdf]

nature portfolio

Corresponding author(s): Chenlong Li

Last updated by author(s): Jan 7, 2024

Reporting Summary

Nature Portfolio wishes to improve the reproducibility of the work that we publish. This form provides structure for consistency and transparency in reporting. For further information on Nature Portfolio policies, see our Editorial Policies and the Editorial Policy Checklist.

Statistics

For all statistical analyses, confirm that the following items are present in the figure legend, table legend, main text, or Methods section.

n/a	Confirmed	
☐	☒	The exact sample size (*n*) for each experimental group/condition, given as a discrete number and unit of measurement
☐	☒	A statement on whether measurements were taken from distinct samples or whether the same sample was measured repeatedly
☐	☒	The statistical test(s) used AND whether they are one- or two-sided<br>*Only common tests should be described solely by name; describe more complex techniques in the Methods section.*
☒	☐	A description of all covariates tested
☒	☐	A description of any assumptions or corrections, such as tests of normality and adjustment for multiple comparisons
☐	☒	A full description of the statistical parameters including central tendency (e.g. means) or other basic estimates (e.g. regression coefficient) AND variation (e.g. standard deviation) or associated estimates of uncertainty (e.g. confidence intervals)
☐	☒	For null hypothesis testing, the test statistic (e.g. *F*, *t*, *r*) with confidence intervals, effect sizes, degrees of freedom and *P* value noted<br>*Give P values as exact values whenever suitable.*
☒	☐	For Bayesian analysis, information on the choice of priors and Markov chain Monte Carlo settings
☒	☐	For hierarchical and complex designs, identification of the appropriate level for tests and full reporting of outcomes
☐	☒	Estimates of effect sizes (e.g. Cohen's *d*, Pearson's *r*), indicating how they were calculated

Our web collection on statistics for biologists contains articles on many of the points above.

Software and code

Policy information about availability of computer code

Data collection	For RNA-seq/ChIP-seq/ATAC-seq/MNase-seq: Novaseq;<br>For IP-MS: Thermo Scientific Ultimate 3000 UHPLC couped with Q Exactive HF;<br>For microscopy: Zeiss LSM800;<br>For growth phenotypes: Canon EOS 80D(W) camera;<br>For qPCR: StepOne Plus (Applied Biosystems);
Data analysis	RNA-seq analysis: Salmon version 1.5.1, DESeq2 version 3.1.0<br>ChIP-seq/ATAC-seq analysis: Bowtie for Illumina version 1.1.2, SAMTools version 3.3.0.0.0, MACS2 version 1.1.2, DeepTools version 2, Integrative Genomics Viewer (version 2.4.14), ChIPseeker Version 1.18.0+galaxy1, DiffBind version 2.10.0<br>MNase-seq: cutadapt (version 3.4, -m 10), Bowtie2,DANPOS3, SAMTools version 3.3.0.0.0<br>GO analysis: DAVID Bioinformatics Resources (https://david.ncifcrf.gov/) and HIPLOT (hiplot-academic.com).<br>Phylogenetic analysis: MEGA11<br>Confocol microscopy: LSM880 with Fast airy scan, Zen<br>Western blot: Molecular Imager ChemiDoc XRX+(BIO-RAD); Image lab 5.0 and e-Blot Touch Image<br>IP-MS: Proteome Discoverer 2.4.1.15 (IP-MS data URL:https://www.iprox.cn/page/SSV024.html;url=1696754351230fA7E; Password: fnmY)<br>Others: ImageJ (1.50i), BioVenn (2007), Excel (2016), StepOne Software (v2.3)

For manuscripts utilizing custom algorithms or software that are central to the research but not yet described in published literature, software must be made available to editors and reviewers. We strongly encourage code deposition in a community repository (e.g. GitHub). See the Nature Portfolio guidelines for submitting code & software for further information.

Data

Policy information about availability of data

All manuscripts must include a data availability statement. This statement should provide the following information, where applicable:

- Accession codes, unique identifiers, or web links for publicly available datasets
- A description of any restrictions on data availability
- For clinical datasets or third party data, please ensure that the statement adheres to our policy

The ChIP-seq and MNase-seq datasets have been deposited in the Gene Expression Omnibus under accession no. GSE215295 (https://www.ncbi.nlm.nih.gov/geo/query/acc.cgi?acc=GSE215295).
The RNA-seq datasets have been deposited in the Gene Expression Omnibus under accession no. GSE215151 (https://www.ncbi.nlm.nih.gov/geo/query/acc.cgi?acc=GSE215151).
The ATAC-seq datasets have been deposited in the Gene Expression Omnibus under accession no. GSE252623 (https://www.ncbi.nlm.nih.gov/geo/query/acc.cgi?acc=GSE252623).
The mass spectrometry proteomics data have been deposited in the iProX database under the dataset identifier IPX0005203001 (https://www.iprox.cn//page/subproject.html?id=IPX0005203001).
The BRM, H4K5ac, BRD1, BRD2, and BRD13 ChIP-seq data were downloaded from GEO under accession no. GSE161595. BRIP1 and BRIP2 ChIP-seq data were downloaded from GEO under accession no. GSE142369. The H3K27me3 ChIP-seq data were downloaded from GEO under accession no. GSE145387. The SYD and MINU ChIP-seq data were downloaded from GEO under accession no. GSE218841. The brm, syd, and minu ATAC-seq data used in Extended Data Fig. 10 were downloaded from GEO under accession no. GSE193397. The WT ATAC-seq data used in Extended Data Fig. 8a-e were downloaded from Beijing Institute of Genomics Data Center under BioProject PRJCA002620.

Research involving human participants, their data, or biological material

Policy information about studies with human participants or human data. See also policy information about sex, gender (identity/presentation), and sexual orientation and race, ethnicity and racism.

Reporting on sex and gender	N/A
Reporting on race, ethnicity, or other socially relevant groupings	N/A
Population characteristics	N/A
Recruitment	N/A
Ethics oversight	N/A

Note that full information on the approval of the study protocol must also be provided in the manuscript.

Field-specific reporting

Please select the one below that is the best fit for your research. If you are not sure, read the appropriate sections before making your selection.

☒ Life sciences ☐ Behavioural & social sciences ☐ Ecological, evolutionary & environmental sciences

For a reference copy of the document with all sections, see nature.com/documents/nr-reporting-summary-flat.pdf

Life sciences study design

All studies must disclose on these points even when the disclosure is negative.

Sample size	All sample sizes were noted in relevant figure legends or materials and methods.<br>1. For phenotypic analysis, seeds were sown on a mixture of soil and vermiculite (1:1). Seedlings were grown under cold white light (approximately 120 µmol·m-2·s-1) with short-day conditions (10 h light/14 h dark) at 22 °C. Abaxial trichomes were observed with a stereomicroscope 3-4 weeks after planting.<br>2. For leaf shape analysis, fully expanded leaves were removed, attached to cardboard with double-sided tape, flattened with transparent tape, and then scanned in a digital scanner.<br>3. Total RNA was extracted from 14-day-old Arabidopsis seedlings grown under short-day conditions.<br>4. For GUS staining, 14-day-old Arabidopsis seedlings grown under short-day conditions were collected.
Data exclusions	No data exclusion
Replication	ChIP-qPCR, RT-qPCR, RNA-seq:Three biological replicates were used;<br>IP-MS, ChIP-seq, ATAC-seq, MNase-seq: Two biological replicates were used;<br>All attempts at replication were successful.

Randomization	All plant were grown in the same condition and plants were then randomly collected for experiments.
Blinding	Experiments were not blinded. Data were always collected according to the genotype of plants.

Reporting for specific materials, systems and methods

We require information from authors about some types of materials, experimental systems and methods used in many studies. Here, indicate whether each material, system or method listed is relevant to your study. If you are not sure if a list item applies to your research, read the appropriate section before selecting a response.

Materials & experimental systems

n/a	Involved in the study	
☐	☒	Antibodies
☒	☐	Eukaryotic cell lines
☒	☐	Palaeontology and archaeology
☒	☐	Animals and other organisms
☒	☐	Clinical data
☒	☐	Dual use research of concern
☐	☒	Plants

Methods

n/a	Involved in the study	
☐	☒	ChIP-seq
☒	☐	Flow cytometry
☒	☐	MRI-based neuroimaging

Antibodies

Antibodies used	anti-GFP (Abcam, Cat#ab290, Lot#GR3196305-1), Anti-FLAG Magnetic Beads (Sigma, Cat#M8823); anti-FLAG (Sigma, Cat# A8592); Anti-HA-Agarose antibody (Sigma, Cat#A2095, Lot#048M4893V), Anti-HA antibody (Sigma, Cat#H6533), Anti-GFP beads(KT Health, Cat# KTSM1301), and anti-H3 (Proteintech, Cat #:17168-1-AP), anti-GST (TransGen Biotech, Cat#HT601-01), anti-MBP (TransGen Biotech, Cat#HT701-1)
Validation	1. Anti-GFP antibody - ChIP Grade (ab290) is a highly versatile antibody that gives a stronger signal than other anti-GFP antibodies available. Suitable for: Flow Cyt, ELISA, ICC/IF, ChIP, IHC-FrFl, ChIP/Chip, IHC-Wholemount, Electron Microscopy, IHCFoFr, ICC, IHC-P, IHC-Fr, IP, WB. The anti-GFP antibody was validated in Li et al., 2016 Nature Genetics.<br>2. Anti-FLAG Magnetic Beads (Sigma, Cat#M8823) provide an easy, fast and convenient method for the detection and capture of fusion proteins with the FLAG® peptide sequence. ANTI-FLAG M2 Magnetic Beads were validated in https://www.sigmaaldrich.com/catalog/product/sigma/m8823?lang=zh&region=CN.<br>3. anti-FLAG (Sigma, Cat# A8592) was validated in Qi et al., 2017 Plant Cell.<br>4. Anti-HA-Agarose antibody is the immunoglobulin fraction of Monoclonal Anti-HA (mouse IgG1 isotype) covalently linked to agarose. It is suitable for immunoprecipitation detection and immunoaffinity purification of HA labeled fusion protein.<br>5. Anti-HA antibody (Sigma, Cat#H6533) was validated in Qi et al., 2017 Plant Cell.<br>6. Anti-GFP beads (KT Health, Cat# KTSM1301) was validated in Fu et al., 2023 Plant Cell and https://www.ktsm-life.com/Product/info_itemid_230_lcid_249.html.<br>7. anti-H3 (Histone-H3 Rabbit Polyclonal antibody) was validated in http://www.ptgcn.com/products/Histone-H3-Antibody-17168-1-AP.html.<br>8.anti-GST (TransGen Biotech, Cat#HT601-01) was validated in https://www.transgen.com/antibody_tag/388.html.<br>9.anti-MBP (TransGen Biotech, Cat#HT701-1) was validated in https://www.transgen.com/antibody_tag/389.html.

Dual use research of concern

Policy information about dual use research of concern

Hazards

Could the accidental, deliberate or reckless misuse of agents or technologies generated in the work, or the application of information presented in the manuscript, pose a threat to:

No	Yes	
☒	☐	Public health
☒	☐	National security
☒	☐	Crops and/or livestock
☒	☐	Ecosystems
☒	☐	Any other significant area

Experiments of concern

Does the work involve any of these experiments of concern:

No	Yes	
☒	☐	Demonstrate how to render a vaccine ineffective
☒	☐	Confer resistance to therapeutically useful antibiotics or antiviral agents
☒	☐	Enhance the virulence of a pathogen or render a nonpathogen virulent
☒	☐	Increase transmissibility of a pathogen
☒	☐	Alter the host range of a pathogen
☒	☐	Enable evasion of diagnostic/detection modalities
☒	☐	Enable the weaponization of a biological agent or toxin
☒	☐	Any other potentially harmful combination of experiments and agents

Plants

Seed stocks	Transfer DNA insertion lines, bcl7a-1 (SALK_027934) and bcl7b-1 (SALK_029285) were obtained from the Arabidopsis Biological Resource Center (ABRC) and were described recently. Mutants brm-1 (SALK_030046) and brm-3 (SALK_088462) and pBRM:BRM-GFP brm-1 transgenic plants were previously described. The spl9-4 spl15-1 (SAIL_150_B05 and SALK_074426), p35S:MIM156, and pMIR156A:GUS seeds were provided by Pro. Gang Wu (Zhejiang Agriculture and Forestry University) and previously described.
Novel plant genotypes	To construct pBCL7A:BCL7A-GFP and pBCL7B:BCL7B-GFP vectors, the full-length genomic DNA of BCL7A or BCL7B (without the stop codon) containing its corresponding promoter sequence (~2.7 kb for BCL7A promoter and ~2.3 kb for BCL7B promoter) was amplified from genomic DNA by PCR, cloned into the pDONR221 vector by BP reaction (Invitrogen,), and further subcloned into the destination plasmid pMDC10773 by LR reaction (Invitrogen). The constructs were introduced into Agrobacterium tumefaciens strain GV3101 and were then used to transform bcl7a bcl7b double mutant plants using the floral dip method. The pBCL7A:BCL7A-GFP bcl7a was isolated after crossing pBCL7A:BCL7A-GFP bcl7a bcl7b with brm-1. The same strategy was used to obtain pBCL7B:BCL7B-GFP bcl7b. To construct the vector overexpressing MIR156A precursors, a genomic DNA fragment containing pre-MIR156A was amplified from genomic DNA by PCR as previously described24, and further subcloned into a modified overexpression vector pFGC-UBQ10pro75 using ClonExpress II One Step Cloning Kit (Vazyme Biotech, Cat. No. C112). The construct was introduced into Agrobacterium tumefaciens strain GV3101 and were then used to transform WT and bcl7a bcl7b double mutant plants using the floral dip method. Primers used for constructing are listed in Supplementary Table 4.
Authentication	*Describe any authentication procedures for each seed stock used or novel genotype generated. Describe any experiments used to assess the effect of a mutation and, where applicable, how potential secondary effects (e.g. second site T-DNA insertions, mosiacism, off-target gene editing) were examined.*

ChIP-seq

Data deposition

☒ Confirm that both raw and final processed data have been deposited in a public database such as GEO.

☒ Confirm that you have deposited or provided access to graph files (e.g. BED files) for the called peaks.

Data access links<br>*May remain private before publication.*	https://www.ncbi.nlm.nih.gov/geo/query/acc.cgi?acc=GSE215295
Files in database submission	GSM6631336 ChIP_seq_BCL7A_IP1<br>GSM6631337 ChIP_seq_BCL7A_IP2<br>GSM6631338 ChIP_seq_BCL7A_brm-1_IP1<br>GSM6631339 ChIP_seq_BCL7A_brm-1_IP2<br>GSM6631340 ChIP_seq_BCL7B_IP1<br>GSM6631341 ChIP_seq_BCL7B_IP2<br>GSM6631342 ChIP_seq_BCL7B_brm-1_IP1<br>GSM6631343 ChIP_seq_BCL7B_brm-1_IP2<br>GSM6631344 ChIP_seq_BRM_IP1<br>GSM6631345 ChIP_seq_BRM_IP2<br>GSM6631346 ChIP_seq_BRM_bcl7ab_IP1<br>GSM6631347 ChIP_seq_BRM_bcl7ab_IP2<br>GSM7658933 ChIP-seq_BRM-GFP brm-1_Drosophila<br>GSM7658934 ChIP-seq_BRM-GFP brm-1 bcl7ab_Drosophila
Genome browser session<br>(e.g. UCSC)	http://www.igv.org/ (IGV_Win_2.4.14)

Methodology

Replicates	Two biological replicates
Sequencing depth	For each ChIP-seq sample, about 5G raw data were abtained.
Antibodies	anti-GFP, Abcam, Cat#ab290, Lot#GR3196305-1
Peak calling parameters	MACS2 was used for peak calling using the following parameters: Paired-end; Effective genome size=119,667,750; nomodel; extension size=200; q value=0.05.
Data quality	In brief, raw data was trimmed by fastp with following parameters: "-g -q 5 -u 50 -n 15 -l 150". The clean data was mapped to the A. thaliana reference genome (TAIR10) using Bowtie 2 with the default settings. Only perfectly and uniquely mapped reads were used for further analysis. A summary of the number of reads for each sample is given in Supplementary Table 5.
Software	ChIP-seq analysis: Bowtie2 version 2.4.5, SAMTools version 3.3.0.0.0, MACS2 version 1.1.2, DeepTools version 2, Integrative Genomics Viewer (version 2.4.14), ChIPseeker Version 1.18.0+galaxy1, DiffBind version 2.10.0
